# Operationalization of critical care triage during a pandemic surge using protocolized communication and integrated supportive care

**DOI:** 10.1186/s40560-020-00475-y

**Published:** 2020-08-06

**Authors:** Devanand Anantham, Crystal Chai-Lim, Jamie Xuelian Zhou, Ghee Chee Phua

**Affiliations:** 1 Duke-NUS Medical School, Singapore, Singapore; 2 Department of Respiratory and Critical Care Medicine, Singapore General Hospital, Academia Building Level 3, 20 College Road, Singapore, S169856 Singapore; 3 Medical Social Services Department, Singapore General Hospital, Singapore, Singapore; 4 Division of Supportive and Palliative Care, National Cancer Centre Singapore, Singapore, Singapore

**Keywords:** COVID-19, Communication, Ethics, Intensive care unit, Pandemic surge, Supportive care, Triage

## Abstract

Triage becomes necessary when demand for intensive care unit (ICU) resources exceeds supply. Without triage, there is a risk that patients will be admitted to the ICU in the sequence that they present, disadvantaging those who either present later or have poorer access to healthcare. Moreover, if the patients with the best prognosis are not allocated life support, there is the possibility that overall mortality will increase. Before formulating criteria, principles such as maximizing lives saved and fairness ought to have been agreed upon to guide decision-making. The triage process is subdivided into three parts, i.e., having explicit inclusion/exclusion criteria for ICU admission, prioritization of patients for allocation to available beds, and periodic reassessment of all patients already admitted to the ICU. Multi-dimensional criteria offer more holistic prognostication than only using age cutoffs. Appointed triage officers should also be enabled to make data-driven decisions. However, the process does not merely end with an allocation decision being made. Any decision has to be sensitively and transparently communicated to the patient and family. With infection control measures, there are challenges in managing communication and the psychosocial distress of dying alone. Therefore, explicit video call protocols and social services expertise will be necessary to mitigate these challenges. Besides symptom management and psychosocial management, supportive care teams play an integral role in coordination of complex cases. This scoping review found support for the three-pronged, triage-communication-supportive care approach to facilitate the smooth operationalization of the triage process in a pandemic.

## Background

By 7 July 2020, there have been 11,272,342 COVID-19 infections diagnosed worldwide with 531, 056 deaths and approximately 200,000 new cases reported daily [1]. Surges in COVID-19 patients with respiratory failure have already overwhelmed intensive care unit (ICU) resources in Europe, forcing frontline staff to make rationing decisions on which patients were offered life support [2]. Building on these lessons and in anticipation of an increase in demand for critical care, national and institutional measures have been instituted. These measures involve freeing up ICU resources by postponing elective procedures, upskilling medical professionals to manage critical care, and mobilizing national reserves of ventilators [3]. Nevertheless, with an anticipated symptomatic attack rate of 30% in the community and of these patients, 5 to 8% possibly requiring ICU admission, it is prudent to operationalize triage procedures should the demand for critical care exceed capacity [4].

Without a triage plan, there is a risk that patients will be admitted to the ICU in the sequence that they present. This will unfairly disadvantage patients who either present later in the pandemic or have poorer access to healthcare [5]. Younger COVID-19 patients without cardiovascular co-morbidities have a better prognosis [6, 7], and if they should present later when ICU resources are already depleted by allocation to earlier patients who happened to have a poorer prognosis, then we risk increasing overall mortality. Moreover, subjective individual decision-making at the bedside results in both inconsistent decisions and moral distress among healthcare professionals. This distress causes burnout that is ill afforded in the midst of a pandemic [8]. Three areas have been identified where reconfigured processes are needed for a pandemic surge: triage, communication with patients, and supportive care.

1. Triage: This is a complex process, and producing an algorithm is but the first step. Fixed criteria will not be helpful because local surges in patient numbers and availability of resources are in constant flux. Resource calculations cannot be limited to only ICU beds or ventilators. In a global crisis, with multiple national lockdowns and disrupted supply chains, just-in-time economic models have also resulted in shortage of supplies of drugs (e.g., analgesic and paralytic agents) and consumables (e.g., ventilator filters and tubing) [9]. Therefore, triage needs to be adaptable while concurrently anchored in substantive principles. In addition, data-driven deliberations improve quality of decision-making by minimizing bias.

(2) Communications: At the bedside, triage does not merely end with an allocation decision being made. Any decision has to be sensitively and transparently communicated to the patient and family. With infection control measures heightened and isolation protocols enforced, there are unique challenges in effectively managing communication. Video calls offer both opportunities and challenges that clinical teams may be unfamiliar with. Therefore, explicit communication protocols and social services expertise will be necessary to mitigate these challenges.

(3) Supportive care: There remains a duty to care for patients who are not allocated ICU resources. Upgrading general ward facilities to offer acute non-invasive ventilation or high flow oxygen therapy can provide respiratory support to some of these patients. Awake prone positioning is also being investigated [10, 11]. Nevertheless, many patients will require palliation and end-of-life care that ought not be an afterthought. Patients who have already been admitted to the ICU but on re-assessment found to be unsuitable for continued life support will also fall into this category. Moreover, infection control measures create unique challenges such as the psychosocial distress of dying alone. Early referral to supportive care services with concurrent rather than sequential provision of palliation is preferred because resuscitative care and symptom control are not mutually exclusive [12, 13]. Supportive care plays the additional role of coordinating multidisciplinary efforts and comforting distressed frontline staff [14].

Our scoping review of the literature has resulted in a recommendation for the creation of a three-pronged approach. The approach involves the actual critical care triage process by independent triage officers, formulating protocolized communications and early referrals for supportive care interventions. Critical care triage requires both explicit criteria for allocation of ICU resources, as well as, a workflow that specifies who will be responsible for allocation, when their input is required and how these decisions will be audited. Any communication protocol must balance the sensitivity of conveying difficult decisions and infection control requirements. Supportive care offers symptom management, psychosocial support, and facilitation of interprofessional coordination. Our review has also identified areas for further research that can inform future decision-making.

## Critical care triage

**Fig. 1:**
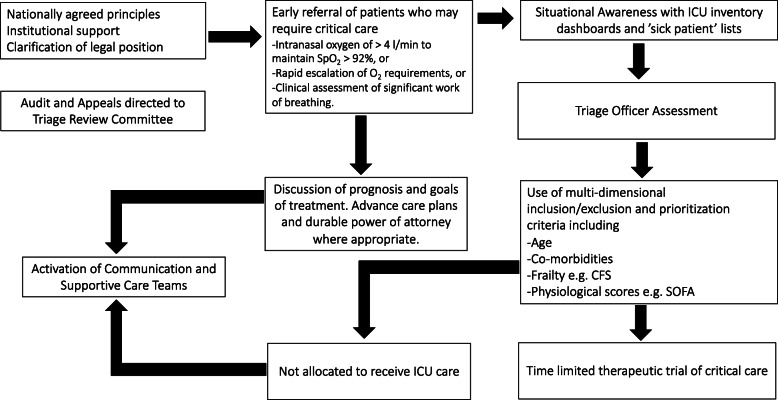
Triage workflow. CFS, Clinical Frailty Score; SOFA, sequential organ failure assessment

The triage workflow is designed to proceed in a sequential manner when ICU demand exceeds capacity (Fig. 1). First, principles to guide decision-making ought to have been agreed upon. Then, multi-dimensional triage criteria are employed to facilitate prognostication and prioritize use of resources. Appointed triage officers are enabled to make data-driven decisions and an audit, as well as appeal mechanism is established. These measures ensure that the entire workflow is guided by procedural values of reasonableness, transparency, inclusiveness, responsiveness, and accountability [4].

Triage criteria: The principles of the triage process ought to be guided by nationally agreed ethical standards such as saving as many lives as possible and using the same allocation criteria on all patients without discrimination. The diagnosis of COVID-19 should not itself confer either a positive or negative bias on any patient’s assessment, and all should be assessed equally [5]. These values are not adopted as merely self-justifying, prima facie obligations, but also provide triage officers with protection should there be any legal challenge. Adherence to nationally agreed guidelines can be considered as strong evidence of fulfilling the standard of care in medical practice [15]. In addition, these principles are consistent with international recommendations such as those of the United Nations Educational, Scientific, and Cultural Organization (UNESCO) International Bioethics Committee, “Macro- and micro-allocation of healthcare resources are ethically justified only when they are based on the principle of justice, beneficence, and equity.” [16].

Patients arriving for triage can essentially be categorized as either being too well and not requiring critical care, or unlikely to survive regardless of ICU provision, or sick enough and likely to benefit from critical care [4]. Triage is essentially about accurately identifying and admitting patients in the third category. Criteria for triage should be based on likelihood of both benefit and need [17]. To meet these ends, the triage process is subdivided into three parts, i.e., having explicit inclusion/exclusion criteria for ICU admission, prioritization of patients for allocation to available beds, and periodic reassessment of all patients already admitted to the ICU [18].

Age has been shown to be a negative prognostic factor for mortality in COVID-19 [19, 20]. Nevertheless a proportion of elderly patients have survived and have been discharged after ICU treatment [17, 21]. Because aging is a heterogeneous process and has imperfect correlation with comorbidities [22], efforts have been made to avoid triaging in a unidimensional manner using only age cutoffs. The National Institute for Health and Care Excellence has advocated the use of the Clinical Frailty Scale with a cutoff of ≥ 5 (mildly frail or worse) as a tool that identifies poor outcomes [23]. This scoring system has the advantage of ease of use without compromising predictive ability [24]. In addition, physiological scores such as sequential organ failure assessment (SOFA) have been used to gage expected mortality. However, such scores were developed for use in sepsis, and utility of predicting outcomes in viral pneumonia remains controversial [25, 26]. This underscores the pressing need for research to provide better prognostication of COVID-19 patients.

Triage workflow: The appointment of triage officers ensures consistency in allocation decisions, and to avoid bias, they should have no direct responsibility for patient care [27]. These officers need situational awareness through dashboards on ICU resource inventory, as well as lists of patients who are in potential need of critical care [4]. Their role is to apply predetermined triage criteria according to surge level and approve appropriate ICU admissions. Triage officers should command sufficient clinical stature so as to be able to reassess the prognosis of patients and direct intensivists to withdraw life support if criteria are not met.

Before any triage is done, all patients ought to have been given accurate prognostic information about their likelihood of recovery so that preferences regarding goals of treatment can be ascertained and respected. Where appropriate, durable power of attorneys and previous advance care plans have to be identified [28]. To do this effectively without being subject to undue pressure, early referral is necessary. In COVID-19, a reasonable recommendation for this threshold may be intranasal oxygen of > 4 L/min to maintain SpO_2_ > 92%, or the rapid escalation of oxygen requirements, or clinically assessed significant work of breathing. Although officers, who are implementing approved triage processes, need not require the consent of patients to either withhold or withdraw critical care [4], communicating the allocation decision requires sensitivity so as not to increase the distress of the patient or family. Therefore, the activation of triage officers should be coupled with a triggering of communication and supportive care services to assist with end-of-life discussions [29].

The auditing of triage decisions can be performed by a triage review committee. This committee would also hear appeals if there is disagreement between physicians or if the patient/family is unable to accept the triage decision [5]. Besides senior clinicians, ethicists, and nurses [17], members of the lay public can also be included in this committee to provide broad representation. If there is an appeal, the primary role of the committee is to ascertain that the triage criteria was correctly applied [5, 29]. For this reason, if there are any changes to the triage criteria because of emerging data, these changes have to be submitted to the triage review committee and approved in advance of implementation. If equally deserving patients are vying for the same ICU bed, a life cycle calculus can be employed to maximize number of years of life saved [30]. If a further tie-breaker is required, random selection is used to ensure equitable distribution without any discrimination [5]. The expectation is for the triage review committee to return a binding decision expeditiously (e.g., within a day) and the decision to be documented in the clinical records [29]. If patients require immediate ICU care during the course of a review, they can be temporarily supported with the necessary critical care based on the explicit understanding that life support may be withdrawn depending on the committee’s decision. The entire triage process should be trial run using simulated case scenarios to ensure smooth coordination and a common understanding of principles [4]. All deliberations of the triage review committee ought to be subject to oversight (e.g., through the head of the hospital ethics committee) [4].

Our review has identified several gaps in knowledge. SOFA scores may be non-discriminatory in COVID-19 patients presenting with single organ failure (typically respiratory failure), and alternative physiological prognostication models need to be developed. Buy-in is also needed for the equal consideration model especially in a society where there may be significant immigrant populations [31]. This model can be promoted using the principle of solidarity, i.e., because COVID-19 infects indiscriminately, no one is safe until we are all safe. Prioritization based on instrumental value (e.g., healthcare workers who are infected with COVID-19 through the course of their work) should also be openly discussed and consensus forged.

## Protocolized communication

For patients meeting triage criteria, ICU care should only be offered as a time-limited, therapeutic trial without an open-ended promise of life support [29]. By framing ICU treatment as a time-limited trial, expectations of the family are managed should the patient subsequently not meet triage criteria on reassessment. For patients who do not meet inclusion/exclusion criteria, honest explanations of the expected prognosis should be offered and any illusion of choice not encouraged. When health systems are operating under crisis standards [32], some choices are unavailable. This will be unfamiliar to the public who may be used to having the dominant say in treatment decisions. When triage decisions are not concordant with a patient’s wishes, family members may feel that the medical team is abandoning the patient, and strong emotional reactions can be expected [33]. Consistent and compassionate communication will aid family come to terms with triage outcomes and support them. One tool that can be used is the SHARE talking map: **s**howing the guideline, **h**eadlining what it means for the patient, **a**ffirming the care that will be provided, **r**esponding to emotions, and **e**mphasizing that the same rules apply to everyone [34]. Implementing such tools in a crisis and effective listening [35] to concerns of patients/families may stretch frontline medical teams. Medical social workers experienced in end-of-life conversations can lead the communication arm of triage by both serving as the conduit between family and the medical team, and working collaboratively with supportive care services [36].

**Fig. 2:**
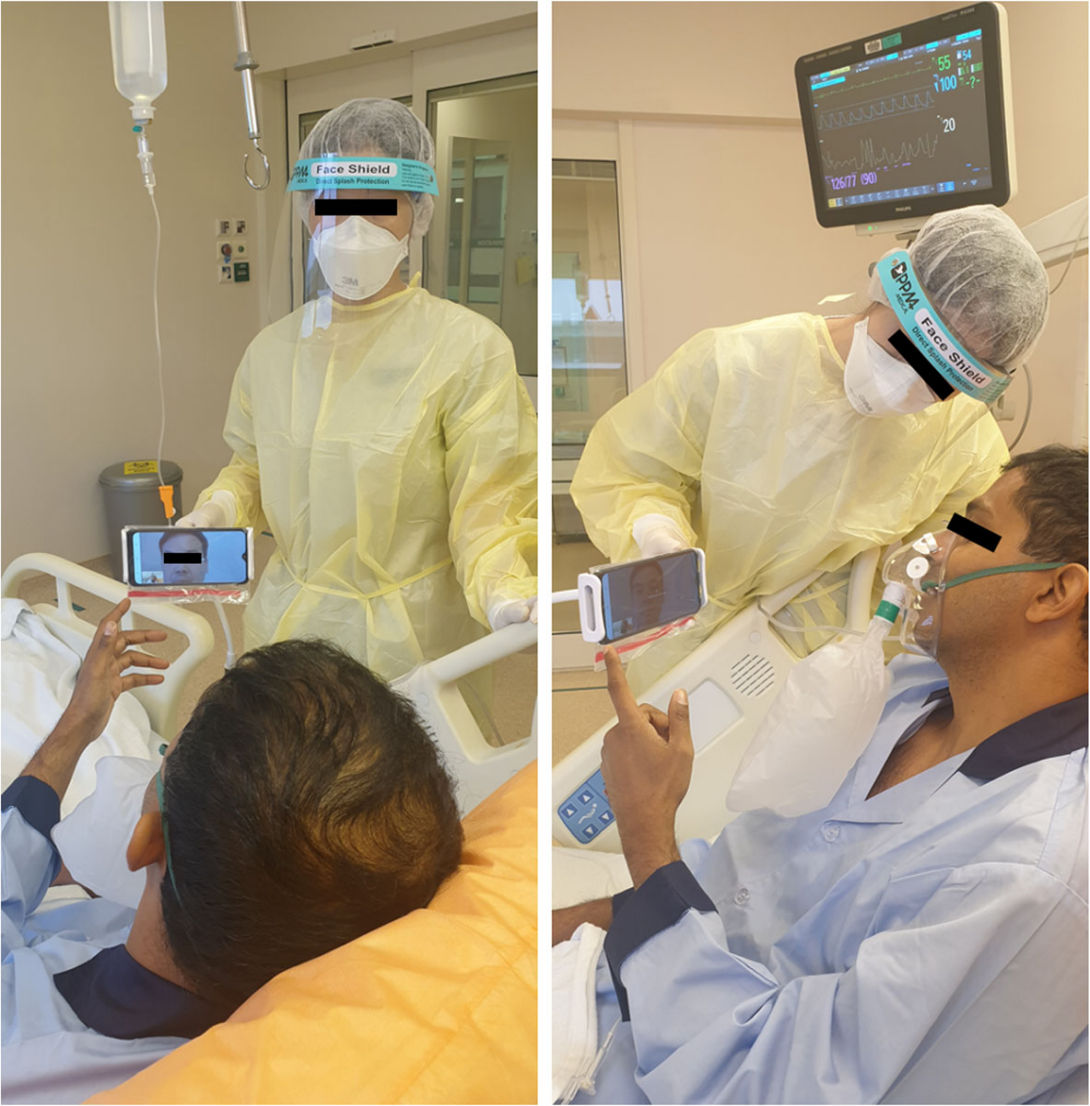
Video call to enable patients in isolation facilities to communicate with family

**Fig. 3:**
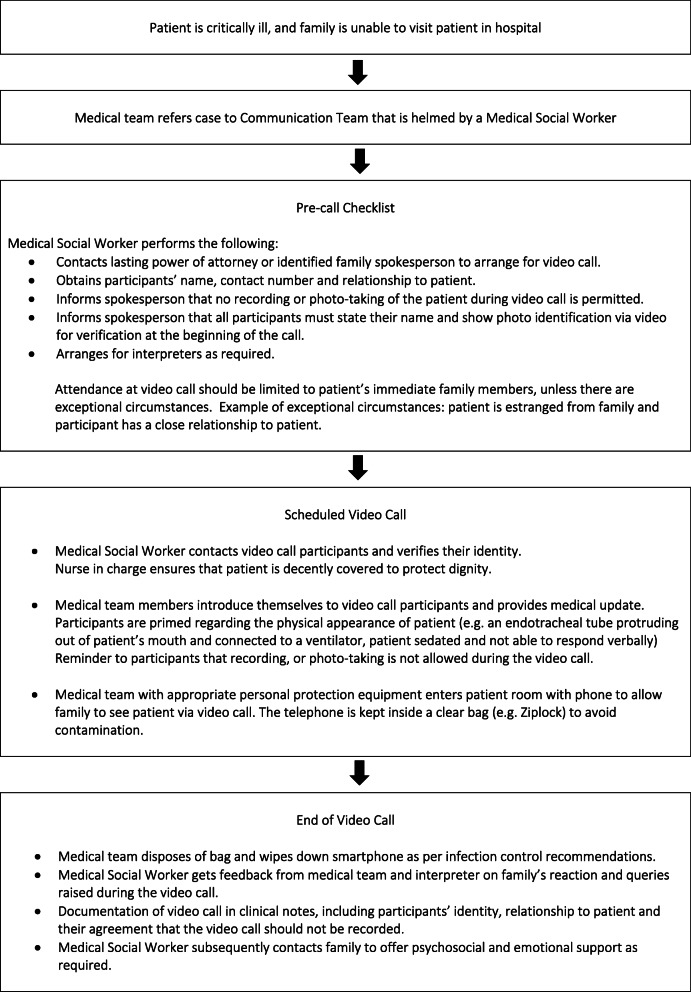
Video call communication protocol

Infection control measures limit face-to-face interactions between ICU teams and the family. Patients with COVID-19 will be isolated, and family members, who had been in close contact, will be unable to visit because of quarantine. Visitation may be only permitted when patients deteriorate to become dangerously ill. Telephone and video calls have become alternatives for connecting the family to the patient. For those who are to ill to make these calls, a protocol can be established for assisted video calls: the nurse enters the patient’s room, and using a dedicated smartphone, lets the family view and speak to the patient (Fig. 2). The family has to be primed about patient’s physical presentation beforehand to minimize distress. This should not be any different from preparing a family before they would physically see a patient admitted to the ICU in usual times outside the pandemic context. Appropriate precautions are necessary to ensure that patient privacy and confidentiality meet both legal and professional standards [37, 38] (Fig. 3). These precautions include ensuring that the patient is decently covered and taking steps to verify the identity of the callers. Similar confidentiality precautions should be undertaken when the ICU team conducts tele or videoconferences with the family for medical updates. Special provisions should also be made for immigrant populations who may face language barriers. These include the availability of interpreters and appropriate translation of information brochures that carry frequently asked questions. The value of these protocolized interventions should be audited through patient and family satisfaction surveys that can be administered after the patient has been discharged.

## Supportive care interventions

Supportive care refers to the holistic management of symptoms and psychosocial issues, the range of services provided not being adequately captured in the term “palliative care.” Palliative care can also carry negative connotations in the minds of patients with a focus on end-of-life and bereavement services. Patients with severe COVID-19 infections need control of multiple symptoms such as dyspnea, cough, fever, and delirium [13]. This will require both non-pharmacological (e.g., facial cooling, relaxation techniques, and breathing exercises) and pharmacological (e.g., opiates, haloperidol, and benzodiazepines) interventions. However, other commonly used techniques such as the use of handheld fans blowing air to the face for relief of dyspnea may not be advisable given the theoretical risk of droplet dispersion [13]. Besides expertise in prescription, supportive care teams also offer conservation strategies that are needed to stretch any limitation in the inventory of available drugs [39]. This includes the use of alternatives such as analgesic suppositories and transcutaneous opiates if supplies of intravenous medications start running low and get prioritized to patients on ventilators.

Data from the severe acute respiratory syndrome epidemic describe complex spiritual and psychosocial issues that can manifest [40]. COVID-19-related respiratory failure warranting long periods of ICU interventions such as prone positioning, paralysis, and sedation can result in post-intensive care syndrome and survivor’s guilt [41, 42].Even without the pandemic situation, family members of patients need to build a trusting alliance with the ICU team, failing which loss of confidence, and dissatisfaction ensues [43]. These family members tend to develop significant sleep disturbance, fatigue, and anxiety [44]. In an isolation ICU, there can be total absence of physical connection between patients and their families due to visitation restrictions. At best, families may end up seeing their loved ones from beyond the glass panels of negative pressure antechambers and are at risk for developing complicated grief if they are unable to support dying patients by the bedside [45, 46]. Proactive engagement mitigates such adverse impact as well as reduces the likelihood of conflict between the ICU team and family [47]. Further data is needed on identifying the highest risk patients/family for psychosocial distress and to assess the effectiveness of interventions on them.

Besides symptom management and psychosocial support, supportive care facilitates interprofessional coordination in complex cases. For ICU survivors, a longitudinal approach to treatment is taken by both linking up with rehabilitation medicine and discharge planning to long-term care facilities. A collection service allows families to deposit personal items to be delivered to their isolated loved ones. These items can include religious resources, photographs, smartphones, and audio recordings to make family members feel engaged in the caregiving. For those who demise, referral to approved funeral directors and specific instructions on how to handle the body of a COVID-19 patient are necessary. Coordination with community partners such as home and inpatient hospice services help to improve right-siting of patients [48]. In addition, appropriate provision of spiritual care and management of staff distress fall within the responsibility of supportive care [14].

Both consultative and integrative models have been described for supportive care services in the ICU [49, 50]. The consultative option is based on involvement of specialized teams who are consulted, and this model has the advantage of dedicated expertise, as well as continuity of care after ICU treatment has been completed. This expertise includes patient-centered decision-making, communication within the team and with families, continuity of care, emotional support for families, and symptom management [51]. The integrative model embeds principles of supportive care in usual ICU management and requires commitment of critical care clinicians to be trained in the necessary skills [50]. These two models are not mutually exclusive, and a mixed model can be employed for the pandemic response.

A co-rounding model has already been shown to result in earlier family meetings and shorter hospital length of stay [52]. During pre-surge preparation, co-rounding facilitates joint assessment of patients with the ICU team to both identify the challenges faced in managing COVID-19 patients and build professional trust. Co-rounding enables efficient interprofessional communication, decision-making for complex cases, and coordination of care. During the surge, there can be a transition to the consultative model with explicit triggers to ensure timely clinical service provision (Table 1). Ultimately, dynamic changes in workflow and clinical load, as well as the unique challenges of this new viral infection, require flexibility and improvisation to provide a commensurate supportive care response [53]. Further research will help identify the most effective model for such a response.

**Table 1:** Indications for referral to specialist supportive care

1. Difficult-to-control physical symptoms despite usual treatment approaches 2. Complex family dynamics impacting decisions about use of life-sustaining treatments 3. Conflicts among staff or between staff and patients/families about prognosis and/or use of life-sustaining treatments 4. Patients/families wanting to explore non-ICU supportive care options such as hospice services 5. Consideration for terminal discharge as defined as discharge from hospital in order to demise at home (in the order of hours to days)	

During the pre-surge period, supportive care teams have taken on the responsibility of creating training resources and guidelines on communicating goals of care, basic symptom management, and end-of-life management [54]. The anticipation is that frontline staff may be deployed to unfamiliar roles, and they will require ready access to support [55, 56]. There is also evidence of considerable variation in withdrawal practices from terminal weaning to immediate extubation [57]. Patients who have been assessed by triage officers to no longer meet criteria for continued life support will need such withdrawal procedures to be performed in a timely manner without causing distress. A terminal extubation checklist with voice-annotated explanations helps by standardizing symptom control algorithms and communication. These checklists, training materials, and guidelines can be made readily accessible through online toolkits [58].

## Conclusions

When demand exceeds supply, triage becomes necessary. If the most appropriate patients with the best chances of survival are not allocated life support, there is the real possibility that overall mortality will increase. Maximizing lives saved and fairness have been the cornerstone of triage criteria. However, the operationalization of such criteria will require real-time data, as well as both communication and supportive care support. Otherwise we may risk paralysis in disposition with inconsistent decisions and multiple demands for appeal from dissatisfied patients and their families. Such paralysis will undermine the triage procedure’s goal of saving lives in a timely manner. The three-pronged, triage-communication-supportive care approach will require multi-disciplinary input and open interprofessional communication for successful implementation that is similar to many other ICU interventions. Protocolized approaches ensure consistency especially in a pandemic where new staff may be working in unfamiliar roles. The complexity of the entire triage procedure will invariably require training, test runs, and iterative refinements as we meet the challenges of COVID-19 head-on.

## Data Availability

Not applicable

## Abbreviations

ICU: Intensive care unit
UNESCO: United Nations Educational, Scientific and Cultural Organization
SOFA: Sequential organ failure assessment
CFS: Clinical Frailty Score
